# Proposal of a Novel Natural Biomaterial, the Scleral Ossicle, for the Development of Vascularized Bone Tissue In Vitro

**DOI:** 10.3390/biomedicines6010003

**Published:** 2017-12-25

**Authors:** Marta Checchi, Jessika Bertacchini, Giulia Grisendi, Alberto Smargiassi, Antonella Sola, Massimo Messori, Carla Palumbo

**Affiliations:** 1 Department of Biomedical, Metabolic Science and Neuroscience, University of Modena and Reggio Emilia, Largo del Pozzo 71, 41125 Modena, Italy; alberto.smargiassi@unimore.it (Al.S.); carla.palumbo@unimore.it (C.P.); 2 Division of Oncology, Department of Medical and Surgical Sciences for Children and Adults, University—Hospital of Modena and Reggio Emilia, Largo del Pozzo 71, 41125 Modena, Italy; giulia.grisendi@unimore.it; 3 Department of Engineering “Enzo Ferrari”, University of Modena and Reggio Emilia, Via Pietro Vivarelli 10/1, 41125 Modena, Italy; antonella.sola@unimore.it (An.S.); massimo.messori@unimore.it (M.M.)

**Keywords:** scleral ossicles, natural scaffold, endothelial cells, osteoblastic cells

## Abstract

Recovering of significant skeletal defects could be partially abortive due to the perturbations that affect the regenerative process when defects reach a critical size, thus resulting in a non-healed bone. The current standard treatments include allografting, autografting, and other bone implant techniques. However, although they are commonly used in orthopedic surgery, these treatments have some limitations concerning their costs and their side effects such as potential infections or malunions. On this account, the need for suitable constructs to fill the gap in wide fractures is still urgent. As an innovative solution, scleral ossicles (SOs) can be put forward as natural scaffolds for bone repair. SOs are peculiar bony plates forming a ring at the scleral-corneal border of the eyeball of lower vertebrates. In the preliminary phases of the study, these ossicles were structurally and functionally characterized. The morphological characterization was performed by SEM analysis, MicroCT analysis and optical profilometry. Then, UV sterilization was carried out to obtain a clean support, without neither contaminations nor modifications of the bone architecture. Subsequently, the SO biocompatibility was tested in culture with different cell lines, focusing the attention to the differentiation capability of endothelial and osteoblastic cells on the SO surface. The results obtained by the above mentioned analysis strongly suggest that SOs can be used as bio-scaffolds for functionalization processes, useful in regenerative medicine.

## 1. Introduction

Bone tissue regeneration is crucial in the orthopedic field. In the past, the most useful treatments for critical-size bone defects resided in filling the gap with autologous bone graft [1]. Recently, the most advanced tissue engineering strategies are addressed to regenerate the impaired tissue using biologically-functionalized constructs. To this aim, not only the optimal construct should be a filler for the bone gap, but it should also mimic the extracellular matrix properties and provide osteogenic stimuli on native bone to trigger/guide the stimulation of the newly-forming tissue in vivo [2]. Inspired by the concept of bone tissue engineering, researchers have been proposing new constructs with osteogenic capabilities (osteoconductive and/or osteoinductive) [3,4]. An essential element for bone regeneration is also the proper vascularization, since the bone tissue is highly-vascularized and the osteogenesis is always coupled with the preliminary growth of blood vessels [5]. Thereby, in order to fill the bone gap, endothelial cells must be induced to colonize inside the newly-forming tissue, thus providing a vascular network, since osteogenic cells need an appropriate platform upon which to produce the bone matrix.

In this study, the broad aim is to characterize a peculiar natural biomaterial, the scleral ossicle (SO), as a support for cell culture, and to test it as a scaffold to heal critical-size bone defects. SOs are dermal bones present in the eyeball of many lower vertebrates with bulging eyes [6,7]; in each chicken eye bulb, 13–14 ossicles form a sclerotic ring located along the scleral-corneal boundary [8]. The functions ascribed to these peculiar ossicles are to support and protect the eyeball against deformation during motion (e.g., flying or swimming) [9,10].

In this preliminary study, the SOs, extracted from the eye bulb of adult chickens, were characterized from a morphological viewpoint and tested for biocompatibility in an in vitro system. The data obtained from this preliminary research pave the way for further experimental investigations focused on the generation of 3D vascularized constructs, usable to generate new bone tissue in critical-size bone defects.

The authors are aware that the SO is very small in comparison to a critical lesion (the average size of calvarial critical defects is about 8 mm in rats [11,12] and 5 mm in mice [13]) but, in the near future, our labs will be involved in the development and manufacturing of engineered 3D-matrixes that can include various SOs in order to fill lesions with critical dimensions.

## 2. Materials and Methods

### 2.1. Scleral Ossicles (SOs) Preparation and Characterization

#### 2.1.1. Extraction and Preparation of SOs

Poultry processing waste, donated by a local butcher, was used as a source of SOs. Each eyeball of adult chickens (50–70 days old) was incised with a scalpel in order to extract the scleral ring. Under a stereomicroscope, the tissue membranes that cover the scleral ring were removed and each of the 13–14 SOs was disjoined. All bony plates were cleaned in PBS (phosphate buffered saline) to eliminate the residues of eye humor liquid, dried in air, and finally sterilized by exposure for 15 min under UV radiations.

#### 2.1.2. Size Calculation

SO thickness was measured using a caliber with a sensibility of 0.01 mm. Ossicle width and length, as well as average dimensions of lacunae and canaliculi were measured by analyzing SEM images with ImageJ software (v1.8.0_101, National Institutes of Health, Bethesda, MD, USA).

#### 2.1.3. Profilometry

Profilometric measurements were performed with a non-contact scanning confocal profilometer (scanning white light) CHR150 (STIL S.A.S, Aix en Provence, France) equipped with an optical pen of 350 μm. Each surface, measuring 300 µm × 100 µm, was scanned by 100 parallel tracings (length = 300 µm) with a point distance of 1 µm. Twelve SOs were analyzed and the corresponding *R_a_* values were calculated by the image elaboration software Gwyddion (v. 2.49 release 15 August 2017, free-software developed by Nečas et al., Brno, Czech Republic). As a term of comparison, the same approach was applied to a human stapes.

#### 2.1.4. Micro Computed Tomography (µCT) Analysis

Micro-CT analysis was performed on three SOs which were scanned with the high-resolution microtomography system Skyscan 1172 (Bruker Micro-CT, Kontich, Belgium) at 60 kV and 170 µA, with a rotation step of 0.3° and a frame averaging of 3. The nominal resolution (pixel size) was set to 2.5 μm. The images obtained from the acquisition were then reconstructed by the software NRecon (Bruker Micro-CT) with corrections for alignment, depending on acquisition, beam hardening correction, and ring artifact reduction maintaining the relative pixel size = 2.5 μm.

Quantitative 3D analyses were carried out with CTAn software (v.1.16.4.1, Bruker Micro-CT). After applying a global threshold, the Volume *V* in mm^3^, the Surface *S* in mm^2^ and the Structure Thickness *St.Th* in mm were calculated.

### 2.2. Scanning Electron Microscopy and X-ray Microanalysis

Samples (four ossicles) were observed with the ESEM Quanta-200 Scanning Electron Microscope (Fei Company, Hillsboro, OR, USA). The samples were fixed for 20 min in 4% paraformaldehyde in PBS (pH 7.4) and dehydrated through a graded series of alcohol (50%, 75%, 95%, 100%) for 10 min each. The specimens were finally dried overnight and coated with gold-palladium (10 nm thickness) using an Emitech K550 sputter coater (Quorum Technologies, South Stour Avenue, Kent, UK). SEM images were collected under low and high vacuum conditions using a large field detector (LFD) and an Everhart Thornley detector (ETD), respectively. Finally, two selected samples were also analyzed via X-ray microprobe (X-ray energy dispersion spectroscopy, X-EDS) using the ESEM software INCA Suite (version 4.07, Oxford Instruments Analytical, Tubney Woods, Abingdon, Oxon, UK) acquiring five spectra for each SO.

### 2.3. Cell Culture

Endothelial Cells (ECs) 1G11 [14], obtained from murine lung and Mouse Aortic Endothelial Cells GFP positive (MAEC GFP^+^), provided by Stefania Mitola (University of Brescia, Brescia, Italy), were cultured in endothelial growth medium composed of Dulbecco’s Modified Eagle’s Medium high glucose with sodium pyruvate (ECB7501L Euroclone, Milano, Italy), supplemented with 10% fetal bovine serum (ECS0180L Euroclone, Milano, Italy).

Osteoblastic cell line 2T3 [15], provided by Dr. Lynda Bonewald (Indiana University School of Medicine, Indianapolis, Indiana), was cultured in complete medium with a composition of Alpha Minimum Essential Medium (AU-L0476-500 Aurogene, Roma, Italy) supplemented with 10% Calf Serum (AU-S0400-500 Aurogene, Roma, Italy).

All media were supplemented with l-glutamine 0.02 mM (ECB3000D Euroclone, Milano, Italy) and 10 U·mL^−1^ penicillin/streptomicin (ECB3001D Euroclone, Milano, Italy).

Confluent cells (80–90%) were sub-cultured routinely at a split ratio of 1:8 three times a week, 1:5 three times a week, and 1:8 two times a week, respectively, for 1G11, MAEC GFP^+^, and 2T3, after trypsin/EDTA digestion and cultured at 37 °C with 5% CO_2_ in a humid atmosphere. Where indicated, cells were differentiated for a well-defined timing in an appropriate culture medium.

### 2.4. Cell Differentiation

In order to better monitor the endothelial differentiation over time, the positive GFP cell line MAEC was used. For endothelial tube formation assay, MAEC GFP^+^ were stimulated using endothelial growth medium added with recombinant Vascular Endothelial Growth Factor (VEGF) at a concentration of 10 ng/mL.

For osteogenic differentiation, a complete medium supplemented with 0.3 mM ascorbic acid and 4 mM β-glycerophosphate was used. The differentiation medium was changed every three days of culture.

Intermediate time points were collected at different times depending on test typology. Each differentiation was performed in triplicate.

### 2.5. Immunofluorescence Analysis

For immunostaining procedure, samples were washed once with PBS followed by fixation in paraformaldehyde 4%/PBS (pH 7.4) for 20 min at room temperature (RT). Fixed specimens were rinsed twice in PBS and then permeabilized in 0.1% Triton X-100/PBS, rinsed twice in PBS, and then saturated in 3% BSA/PBS 1 h at RT. Afterward, samples were incubated with TRITC (tetramethylrhodamine)-conjugated Phalloidin (Millipore 90228, Burlington, MA, USA) diluted 1:500 in 3% BSA/PBS for 1 h at RT, washed three times in PBS, and stained with DAPI (Millipore 90229) for 10 min at RT and washed twice again with PBS. The samples were finally observed with a Nikon Eclipse 80i fluorescent microscope (Nikon Instrument Europe BV, Tripolis 100, Burgerweeshuispad 101, 1076 ER Amsterdam, Amstelveen, The Netherlands).

### 2.6. Trypan Blue Viability Assay

Cells attached to the Petri dish or to SO surfaces were washed with PBS and detached by trypsin/EDTA (Ethylenediaminetetraacetic acid) solution and then resuspended in a known volume of cell medium and counted with trypan blue in mix solution of 1:1 volume. A fraction of this solution (10 µL) was added to the Bürker chamber for the count of the trypan blue negative cells. Triplicate wells were analyzed for each condition and the standard deviation (SD) was calculated.

### 2.7. MTT (3-(4,5-Dimethylthiazol-2-yl)-2,5-Diphenyltetrazolium Bromide) Viability Assay

A total of 9 × 10^4^ cells placed in a 12 multi-well plate were detached after 24 and 48 h and incubated with a final concentration of 0.2 mg/mL MTT solution (5655 Sigma Aldrich, Darmstadt, Germany)/PBS for 3 h at 37 °C. The reaction was blocked adding 100 µL of solubilization solution (10% SDS + 0.01 M HCl/distilled water). The absorbance, at a wave length of 590 and 656 nm, was determined using a Thermo Scientific Appliskan™ plate reader (Thermo Fisher Scientific, 168 Third Avenue, Waltham, MA, USA). Triplicate wells were analyzed for each condition and standard deviation (SD) was calculated.

### 2.8. Ultrastructural Observations

SOs on which 2T3 were differentiated, were fixed for 2 h with 4% paraformaldehyde in 0.13 M phosphate buffer (pH 7.4), postfixed for 1 h with 1% osmium tetroxide in 0.13 M phosphate buffer (pH 7.4), dehydrated in graded ethanol, embedded in epoxy resin (Durcupan ACM; Electron Microscopy Science, Hatfield, PA, USA), and transversely sectioned with a diamond knife mounted in an Ultracut microtome (Reichert-Jung, Wetzlar, Germany). Ultrathin sections (70–80 nm) were mounted on formvar- and carbon-coated copper grids, stained with 1% uranyl acetate and lead citrate, and examined under a Nova NanoSEM 450 (Fei Company, Hillsboro, OR, USA).

### 2.9. Paraffin Inclusion and Immunohistochemistry (IHC)

SOs on which 2T3 were differentiated were fixed for 1 h with 4% paraformaldehyde in 0.13 M phosphate buffer (pH 7.4), rinsed in PBS, decalcified in EDTA 5% for four days, rinsed in PBS and dehydrated through a graded series of alcohol (50%, 70%, 80%, 95%, 100%) for 20 min each. The last incubation was performed in xylene 5 min three times before the inclusion in paraffin. Samples were sectioned in 5 µm thick sections using a Leica RM2255 microtome (Leica, Wetzlar, Germany).

Immunohistochemistry was carried out by deparaffinization of sections for 1 h at 60 °C, incubation of slides in xylene 10 min and a passage through a graded series of alcohol (100%, 95%, 70%, 50%) for 3 min each. Samples were incubated 10 min with a 3% H_2_O_2_ solution prepared in water and rinsed twice in PBS; the antigen retrieval was performed arranging slides in a hot (95–100 °C) sodium citrate buffer (trisodium citrate dehydrate 10 mM) for 10 min; rinsed twice in PBS and treated for 1 h in a blocking buffer (10% FBS (Fetal Bovine Serum)/PBS). Slides were incubated for 16 h with anti-sclerostin primary antibody (ab30044 Abcam, Cambridge, UK) at a concentration of 1:100 in a humidified chamber at 4 °C; after rinsing in PBS, samples were incubated in horseradish peroxidase (HRP)-conjugated secondary antibody for 30 min at RT in a humidified chamber. Finally DAB (3,3′-diaminobenzidine) substrate solution (1:1 DAB-H_2_O_2_ solution) was applied on the sections for 20 min at RT in a humidified chamber. Slides were counterstained by immersion in hematoxylin for 1 min, rinsed in running tap water for 10 min and mounted using DPX mounting solution (06522 Sigma Aldrich, Darmstadt, Germany) after dehydration through a graded series of alcohol (70%, 80%, 95%, 100%) for 5 min each.

### 2.10. Statistical Analysis

Data were expressed as mean ± SD. Differences between experimental conditions were evaluated by Student’s *t*-test. In all analysis, values of *p* < 0.1 were considered statistically significant, while values of *p* > 0.1 were considered highly significant.

## 3. Results

### 3.1. Morphological Characterization

**Figure 1:**
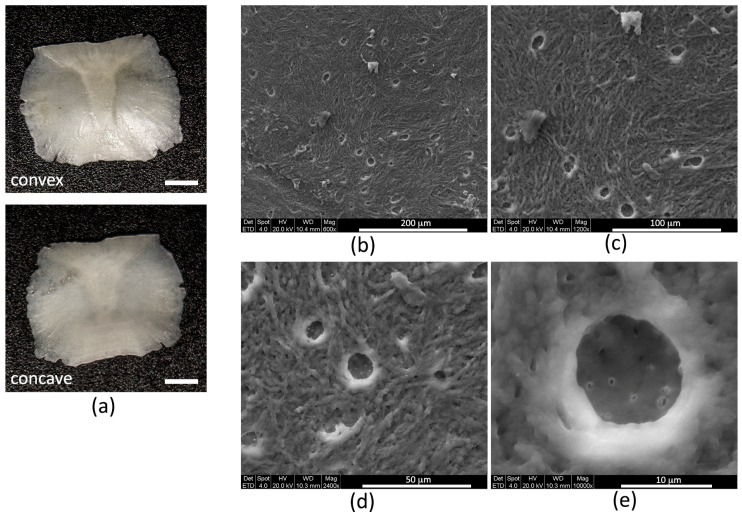
(**a**) Stereomicroscope images of the SO convex and concave surfaces (scale bar = 2 mm); (**b**) SEM image of the SO surface showing osteocyte lacunae; (**c**) enlargement of (**b**); (**d**) SEM (scanning electron microscope) image of osteocyte lacunae; and (**e**) enlargement of (**d**) showing osteocyte canaliculi emerging from an osteocyte lacuna.

Each SO has two sides, one convex and one concave (Figure 1a); average measures are 3 mm in width, 4 mm in length, and 150 µm in thickness (Table 1).

By means of the optical profilometry, it was possible to obtain a value of the surface roughness, expressed in terms of the *R_a_* parameter, which was 1.05 µm (Table 1). For comparative purpose, the same test was repeated on a human stapes, resulting a *R_a_* value of 1.44 µm. The reason for this comparison resides in the analogy of these two types of ossicles, since they are both continuously submitted to stereotyped stresses and strains in vivo [10].

SEM qualitative analysis allowed the identification of the ultrastructural features of the SOs by showing the characteristic presence of woven fibers interposed among numerous osteocyte empty lacunae (Figure 1b–d), where emerging canaliculi are visible (Figure 1e). On SEM images the average size of lacunae and canaliculi are also measurable (Table 1).

Volume (*V*), surface (*S*), and structure thickness (*St.Th*) values, calculated using µCT analysis, are reported in Table 1 and expressed as Average ± SD (Table 1).

**Figure 2:**
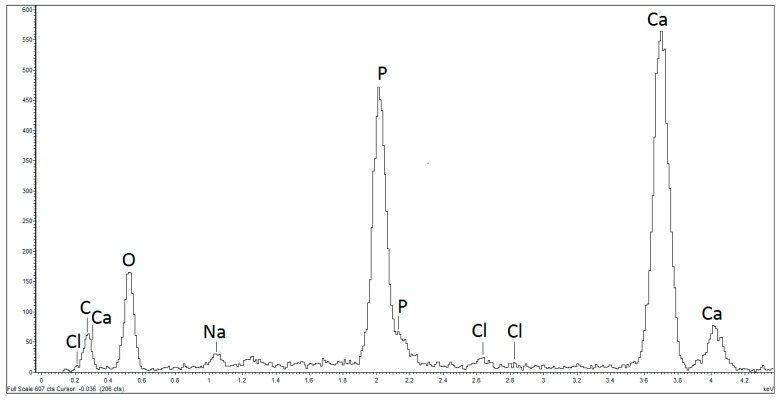
SEM X-ray microprobe semi-qualitative analysis. Ossicle’s surface presents the atoms O, C, Cl, Na, P, and Ca characteristic of the bone tissue.

In addition to the morphological observation of the SO surface under SEM, the atoms present in the first 10 µm of its thickness were analyzed using X-ray microprobe analysis. Figure 2 shows the presence of atoms typical of bone tissue, such as calcium, chlorine, phosphorous, and sodium; the percentage of each element is indicated in Table 2.

### 3.2. Biocompatibility Tests

In order to test the SO biocompatibility in vitro, the behavior of different cell lines seeded on the SO surface were analyzed, performing the experiments in triplicate.

**Figure 3:**
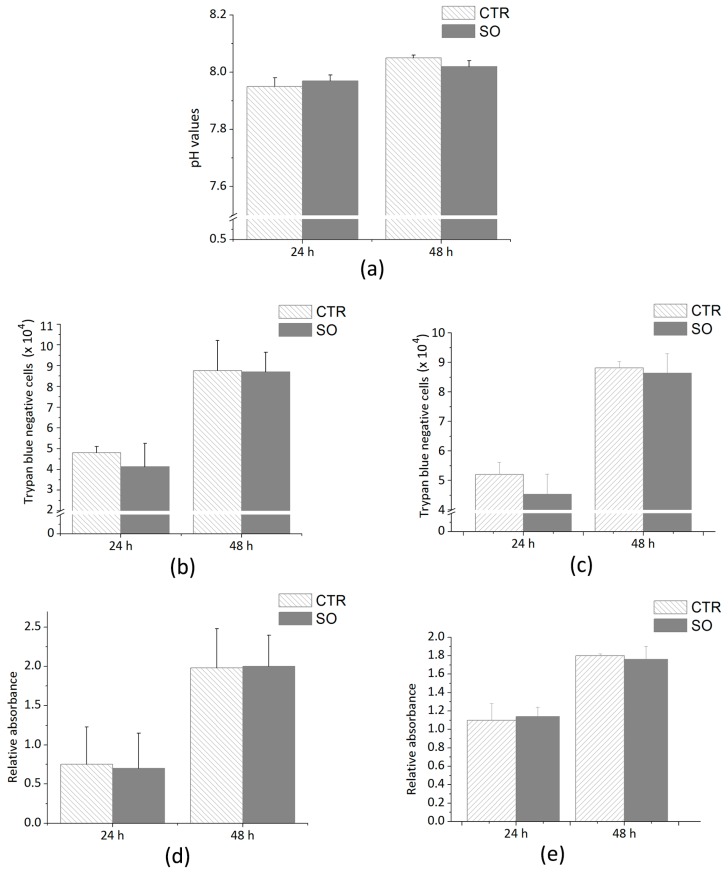
(**a**) pH values of the samples with medium alone (=CTR) or with scleral ossicles (=SO) after 24 h and after 48 h. Number of trypan blue negative cells, 1G11 (**b**) and 2T3 (**c**), counted in samples with (SO) or without (CTR) the scleral ossicles. MTT absorbance related to the number of cells, 1G11 (**d**) and 2T3 (**e**), respectively, counted in samples with (SO) or without (CTR) the scleral ossicles. Data were calculated by three independent experiments and presented as the mean ± SD. *p* > 0.1 is not statistically significant.

Firstly, in order to evaluate the absence of organic soluble compounds released in the culture media following the incubation with SOs, the pH values were measured after 24 and 48 h; the results are shown in Figure 3a. Subsequently, two viability tests were performed on 1G11 and 2T3, namely the trypan blue test and MTT assay. Cells were seeded at a density of 4 × 10^4^ cells in a 24-multiwell plate for 24 ad 48 h with and without SOs. Next, detached cells were stained with trypan blue (1:1) and counted in a Bürker chamber. As shown in Figure 3b,c, the number of cells counted after 24 and 48 h on the plastic plate and SO is similar for both 1G11 and 2T3. Thus, cells were seeded in a 12-multiwell plate at a density of 9 × 10^4^ cells to perform the MTT assay. Cells were grown for 24 and 48 h, either in the presence or in absence of SOs. At the end of the incubation period, MTT assay was performed (Figure 3d,e): the differences between the two experimental conditions are not statistically significant for either 1G11 or 2T3.

### 3.3. Analysis of Cells Adhesion and Proliferation

**Figure 4:**
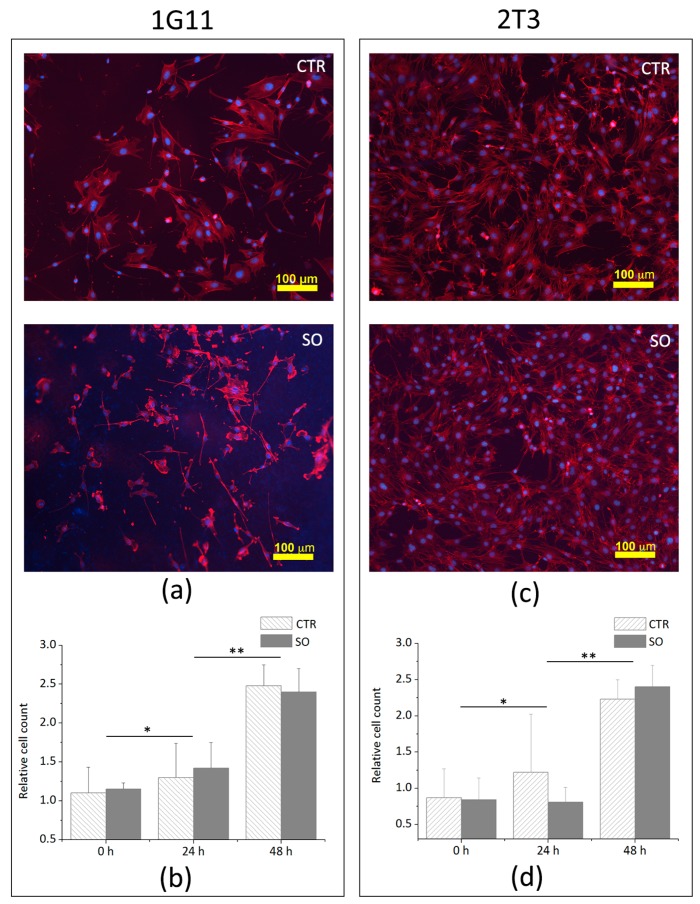
1G11 and 2T3 cultured on plastic plate (CTR) or on scleral ossicle surface (SO); (**a**) morphology of 1G11 cultured for 24 h; (**b**) negative trypan blue 1G11 counted after 24 and 48 h of culture; (**c**) morphology of 2T3 cultured for 24 h; and (**d**) negative trypan blue 1G11 counted after 24 and 48 h of culture (for the immunofluorescence images, nuclei were stained in blue by DAPI, and the cytoskeletons were stained in red by TRITC-conjugated phalloidin). Data in (**b**,**d**) were calculated by three independent experiments and presented as the mean ± SD. * *p* < 0.1; ** *p* < 0.05.

After assessing the biocompatibility of the support, adhesion tests were performed on three different time points in order to verify the possible occurrence of morphological and proliferative changes induced by the bone scaffold. To better understand if cells may undergo morphological changes when seeded onto the ossicle surface, immunofluorescence analyses were carried out; it emerged that the morphology of both cells, 1G11 (Figure 4a) and 2T3 (Figure 4b), is similar to that observed for the cells cultured on plastic plates (Figure 4c).

A proliferation assay was performed on 1G11 and 2T3 plated with 3 × 10^3^ cells on a plastic plate and on the SO surface. For each condition, cells were counted by trypan blue staining upon 24 and 48 h of incubation. Figure 4b,d shows that the number of viable 1G11 and 2T3 cells plated on the SO surface and on the plastic plate are similar, suggesting that the cell proliferation rate is not affected by the SO surface.

### 3.4. Analysis of Endothelial and Osteoblast Differentiation on SO Surfaces

#### 3.4.1. Endothelial Differentiation

**Figure 5:**
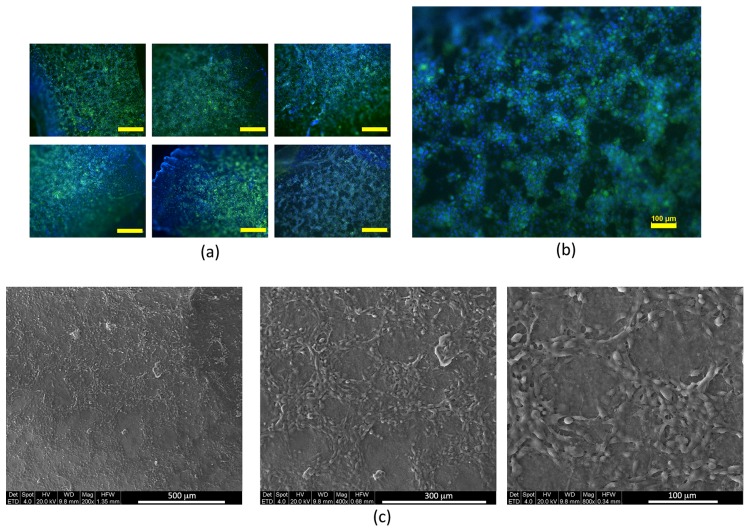
(**a**) MAEC GFP^+^ were seeded on the ossicles and allowed to adhere for 24 h. Then, a treatment with VEGF (10 ng/mL) was performed for 2, 4, and 6 h. It is possible to observe a hint of organization in cords arranged in network after 6 h of stimulation by VEGF (scale bar 500 µm); (**b**) enlargement of the photo at the bottom right in panel (**a**); and (**c**) SEM images of MAEC GFP^+^ stimulated for 6 h with VEGF 10 ng/mL. The same sample shown in (**a**) was prepared and observed under SEM.

Endothelial cells were tested for angiogenic potential using the VEGF-containing medium. MAEC GFP^+^ were seeded on the SO surface for 24 h and subsequently stimulated with VEGF at a concentration of 10 ng/mL for 2, 4, and 6 h. The beginning of organization in a network-like manner was observed after 6 h of stimulation. Figure 5a shows a network-like arrangement where cells progressively organize themselves depending on the time. In order to define the cell arrangement more accurately, SEM observations were performed; as clearly visible in Figure 5c, after 6 h of VEGF stimulation, the cells (mimicking the formation process of vascular buds) showing an arrangement in circular cords that resemble the meshes of a network.

#### 3.4.2. Osteogenic Differentiation

**Figure 6:**
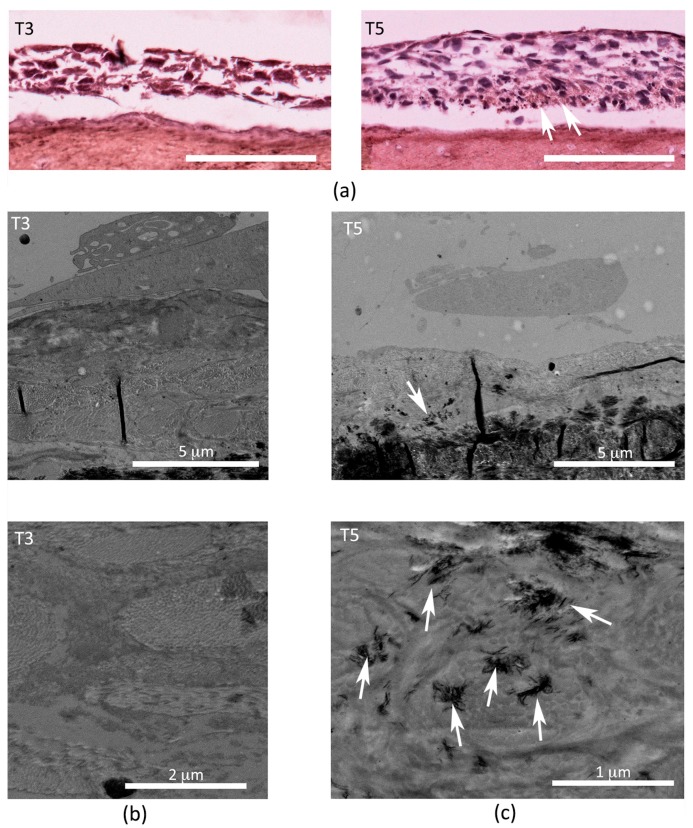
(**a**) Images of Immunohistochemistry (IHC) analysis show a brown staining indicating osteocalcin expression inside the cell layer after five days (T5) of differentiation (white arrows) and absence after three days (T3) of differentiation (scale bar 100 µm). Transmission Electron Microscope (TEM) micrographs showing a transversal section of SO on which 2T3 were differentiated for (**b**) three days and (**c**) five days; top images are enlarged below: it is to be noted that the absence of any calcium deposition in T3 and presence of mineralization dots (white arrows) in T5.

Osteoblast-like cells were tested for osteogenic potential, using medium containing ascorbic acid and β-glicerophosphate. 2T3 were cultured on the SO surface for 24 h in proliferation medium to allow adhesion. At the end of the period, the medium was changed (day 0) with differentiation medium and the conditioning was carried out for 5 days. Intermediate time points were collected at three and five days and ultrastructural and IHC analyses were performed. Osteocalcin expression increases between day 3 and day 5 of differentiation (Figure 6a), and mineralization starts to occur at day 5 (Figure 6b).

## 4. Discussion

In the last decade, important advances in the field of bone repairing have already been achieved: from the direct replacement of defective tissues with first- and second-generation materials (such as bioinert, bioactive [16], and biodegradable materials [17]) to the application of third-generation functionalized materials (materials with the ability to signal and stimulate specific cellular activity and behavior), for the induction of bone self-healing [18]. However, challenging questions still remain unaddressed and the research of new materials is open. Scleral ossicles, that are poultry processing waste, were tested as possible innovative natural biomaterials, at no cost, to use in the generation of 3D constructs useful to improve healing of critical-size bone defects [19]. In this study, various preliminary analyses were performed in order to evaluate firstly the best set-up for the SO biocompatibility and, secondly, the adhesion, proliferation, and differentiation of endothelial and osteoblast-like cells.

The first point to discuss is the suitability of the materials chosen, whose structural features make them similar to other currently used materials, such as decellularized samples from a bone bank. Many researchers, investigating the evolution, have already characterized and described the scleral ossicles [20,21,22,23,24]. Scleral ossicles, present in the eye of many lower vertebrates with bulging eyes [8,25], such as teleosts, birds, and reptiles [26,27], are devoted to restricted functions (i.e., to support and protect the eyeball against deformation during flying or diving [28]). Thus, the functional meaning of scleral ossicles differs from that of the other skeletal segments, since they are submitted to stereotyped strains for all life, and for this reason, after reaching their final size, they do not need (they even need to avoid) mechanical/metabolic adaptation. This is the reason why, in scleral ossicles (like in human auditory ossicles), the mechanism to make the bone structure stable over time, thus maintaining mechanical resistance, is to avoid bone remodeling by means of massive osteocyte programmed death [9]. Indeed, bone remodeling is a process (in response to mechanical and metabolic demands, changing over time) where firstly mature bone tissue is removed by osteoclasts and later new bone tissue is formed by osteoblasts. The osteocytes (i.e., the bone mechano-sensors) have the function to orchestrate, in response to mechanical and metabolic stimuli, the processes occurring in bone remodeling [29,30,31], which are those to be avoided in scleral ossicles [10]; therefore, scleral ossicles represent interesting acellular natural scaffolds. As already mentioned, not irrelevant is the consideration that SOs are poultry processing wastes obtained from the avian food industry and, therefore, they are available in large amounts without expensive production costs. Thus, in this preliminary study, the SOs extracted from the eye bulb of adult chicken, after characterization at the morphological level, were tested for their biocompatibility in in vitro systems. The data obtained confirm the SO suitability and encourage further investigations focused on the generation of 3D vascularized construct.

To check the biocompatibility of the UV-sterilized scaffolds, the pH of the culture medium was analyzed when incubated with and without SOs after 24 and 48 h. The outcomes clearly show that SOs do not release any soluble component that can acidify the pH of the medium. Another point to discuss is that, since 1G11 [32,33,34] and 2T3 [35,36,37] are largely used to mimic, respectively, endothelium development and osteoblast-osteocyte transition in the in vitro experiments, in this study they were used to perform tests of biocompatibility and differentiation. Subsequently, the adhesion, the viability, and the proliferation of both endothelial and osteoblast cells on SOs were analyzed. Trypan blue staining and MTT demonstrated that SOs do not interfere with the normal process of in vitro culture. Finally, to investigate whether SOs affect the differentiation capability of endothelial and osteoblast cells, the tube formation by endothelial cells and the mineralization by osteoblast-like cells were monitored.

As far as osteoblast-like cells are concerned, besides the specific markers of osteogenic differentiation, which are clearly expressed particularly in in vivo environments or in consolidated in vitro models, the occurrence of cell growth, osteocalcin expression increase and mineral crystal deposition are commonly accepted as early demonstrations of the differentiation process of cells seeded on the various scaffolds used in regenerative medicine [38,39,40,41,42]. As far as endothelial cells, the formation of cords arranged in network suggests the undertaken path towards endothelial differentiation.

The proposal here presented agrees also with some recent suggestions by Mercado-Pagán et al. [43] according to whom “to achieve tissue regeneration and integration, cells and signals need to be incorporated into the constructs in a fashion that can establish stable graft-host tissue interfaces, and facilitate instant or rapid blood perfusion across the constructs”. The present paper propounds that the “stable graft-host tissue interface” is likely properly achieved by the vascularized-SO-engineered-construct described here and already presented as a preliminary proposal [44,45].

All the observations here reported suggest that SO scaffolds show promising perspectives in terms of induced pro-angiogenic mechanism and in terms of enhanced osteoblast-like cells and activation of extracellular matrix mineralization processes. Both the capabilities to mimic vascular-like networks and to induce matrix mineralization by cell models are prerequisites to reliably reproduce the bone morphogenetic processes in vitro [46,47]. The differential analyses here performed allow an accurate prediction of the possible behavior of endothelial and osteoblast cells that could be attached to SOs and implanted as 3D-functionalized scaffolds in in vivo critical defects; as a speculative aspect, a possible expected result could be the generation of novel 3D constructs for regenerative medicine.

## 5. Conclusions

These findings suggest that SOs could be used as innovative natural scaffolds: after the extraction from the eye bulb of adult chickens and the functionalization with appropriate cell cultures, they could be useful in the field of the regenerative medicine for the healing of critical-size bone defects. These preliminary results are encouraging in proposing the use of these ossicles as scaffolds on which the ECs are induced to arrange themselves in a microvascular-like network, in turn, conditioning the capability of osteoblast-like cells to produce mineralizing matrix.

## Figures and Tables

**Table 1:** Structural parameters of SOs.

**Size of SOs (mm)**		**Lacuno-Canalicular Size (µm)**	
Length	3.886 ± 0.326	Lacunae	12.790 ± 3.720
Width	3.011 ± 0.334	Canaliculi	0.503 ± 0.222
Thickness	0.16 ± 0.04		
**Roughness *R_a_* (µm)**		**µCT Analysis**	
1.05 ± 0.50		*V* (mm^3^)	1.15 ± 0.31
		*S* (mm^2^)	26.95 ± 3.65
		*St.Th* (mm)	0.13 ± 0.02

**Table 2:** Atoms present on the SO surface expressed in wt%.

C	O	Na	Cl	Ca	P
40.05 ± 3.33	36.28 ± 1.60	0.44 ± 0.07	0.06 ± 0.03	15.48 ± 2.13	7.67 ± 0.68

## References

[1] Harris J.S., Bemenderfer T.B., Wessel A.R., Kacena M.A. (2013). A review of mouse critical size defect models in weight bearing bones. Bone.

[2] Schroeder J.E., Mosheiff R. (2011). Tissue engineering approaches for bone repair: Concepts and evidence. Injury.

[3] Polo-Corrales L., Latorre-Esteves M., Ramirez-Vick J.E. (2014). Scaffold design for bone regeneration. J. Nanosci. Nanotechnol..

[4] Laurencin C., Khan Y., El-Amin S.F. (2006). Bone graft substitutes. Expert Rev. Med. Devices.

[5] Kusumbe A.P., Ramasamy S.K., Adams R.H. (2014). Coupling of angiogenesis and osteogenesis by a specific vessel subtype in bone. Nature.

[6] Coulombre A.J., Coulombre J.L. (1962). The skeleton of the eye. I. Conjunctival papillae and scleral ossicles. Dev. Biol..

[7] Lima F.C., Vieira L.G., Santos A.L.Q., De Simone S.B.S., Hirano L.Q.R., Silva J.M.M., Romao M.F. (2009). Anatomy of the scleral ossicles in Brazilian birds. Braz. J. Morphol. Sci..

[8] Warheit K.I., Good D.A., De Queiroz K. (1989). Variation numbers of scleral ossicles and their phylogenetic transformations within the Pelecaniformes. Auk.

[9] Palumbo C., Presutti L., Genovese E., Cavani F., Sena P., Benincasa M., Ferretti M. (2012). Role of osteocyte apoptosis in peculiar ossicles of the hearing sense organ: Preliminary observations on hearing loss and osteoporosis. Ital. J. Anat. Embryol..

[10] Palumbo C., Cavani F., Sena P., Benincasa M., Ferretti M. (2012). Osteocyte apoptosis and absence of bone remodeling in human auditory ossicles and scleral ossicles of lower vertebrates: A mere coincidence or linked processes?. Calcif. Tissue Int..

[11] Schmitz J.P., Hollinger J.O. (1986). The critical size defect as an experimental model for craniomandibulofacial nonunions. Clin. Orthop. Relat. Res..

[12] Schmitz J.P., Schwartz Z., Hollinger J.O., Boyan B.D. (1990). Characterization of rat calvarial nonunion defects. Acta Anat. (Basel).

[13] Cowan C.M., Shi Y.-Y., Aalami O.O., Chou Y.-F., Mari C., Thomas R., Quarto N., Contag C.H., Wu B., Longaker M.T. (2004). Adipose-derived adult stromal cells heal critical-size mouse calvarial defects. Nat. Biotechnol..

[14] Dong Q.G., Bernasconi S., Lostaglio S., De Calmanovici R.W., Martin-Padura I., Breviario F., Garlanda C., Ramponi S., Mantovani A., Vecchi A. (1997). A General Strategy for Isolation of Endothelial Cells from Murine Tissues: Characterization of Two Endothelial Cell Lines from the Murine Lung and Subcutaneous Sponge Implants. Arterioscler. Thromb. Vasc. Biol..

[15] Ghosh-Choudhury N., Windle J.J., Koop B.A., Harris M.A., Guerrero D.L., Wozney J.M., Mundy G.R., Harris S.E. (1996). Immortalized murine osteoblasts derived from BMP 2-T-antigen expressing transgenic mice. Endocrinology.

[16] Guo B., Lei B., Li P., Ma P.X. (2015). Functionalized scaffolds to enhance tissue regeneration. Regen. Biomater..

[17] Mota C., Puppi D., Chiellini F., Chiellini E. (2015). Additive manufacturing techniques for the production of tissue engineering constructs Carlos. J. Tissue Eng. Regen. Med..

[18] Barabaschi G.D.G., Manoharan V., Li Q., Bertassoni L.E. (2015). Engineering Pre-vascularized Scaffolds for Bone Regeneration. Adv. Exp. Med. Biol..

[19] Spicer P.P., Kretlow J.D., Young S., Jansen J.A., Kasper F.K., Mikos A.G. (2012). Evaluation of bone regeneration using the rat critical size calvarial defect. Nat. Protoc..

[20] Franz-Odendaal T.A., Hall B.K., Witten P.E. (2006). Buried alive: How osteoblasts become osteocytes. Dev. Dyn..

[21] Duench K., Franz-Odendaal T.A. (2012). BMP and Hedgehog signaling during the development of scleral ossicles. Dev. Biol..

[22] Jourdeuil K., Franz-Odendaal T.A. (2012). Vasculogenesis and the Induction of Skeletogenic Condensations in the Avian Eye. Anat. Rec..

[23] Jabalee J., Hillier S., Franz-Odendaal T.A. (2013). An investigation of cellular dynamics during the development of intramembranous bones: The scleral ossicles. J. Anat..

[24] Jabalee J., Franz-Odendaal T.A. (2015). Vascular endothelial growth factor signaling affects both angiogenesis and osteogenesis during the development of scleral ossicles. Dev. Biol..

[25] Franz-Odendaal T.A. (2008). Toward understanding the development of scleral ossicles in the chicken, Gallus gallus. Dev. Dyn..

[26] Edinger T. (1929). Uber knocherne Scleralringe. Zool. Jahrb. Abt. Anat..

[27] Martin G.R. (1985). Form and Function in the Optical Structure of Bird Eyes. Perception and Motor Control in Birds.

[28] Romer A.S. (1956). Osteology of the Reptiles.

[29] Palumbo C., Ferretti M., Ardizzoni A., Zaffe D., Marotti G. (2001). Osteocyte-osteoclast morphological relationships and the putative role of osteocytes in bone remodeling. J. Musculoskelet. Neuronal Interact..

[30] Dallas S.L., Prideaux M., Bonewald L.F. (2013). The osteocyte: An endocrine cell … and more. Endocr. Rev..

[31] Marotti G., Palumbo C. (2007). The mechanism of transduction of mechanical strains into biological signals at the bone cellular level. Eur. J. Histochem..

[32] Viñals F., Pouysségur J. (2001). Transforming Growth Factor β1 (TGF-β1) Promotes Endothelial Cell Survival during In Vitro Angiogenesis via an Autocrine Mechanism Implicating TGF-α Signaling. Mol. Cell. Biol..

[33] Gonzalvo-Feo S., Del Prete A., Pruenster M., Salvi V., Wang L., Sironi M., Bierschenk S., Sperandio M., Vecchi A., Sozzani S. (2014). Endothelial Cell-Derived Chemerin Promotes Dendritic Cell Transmigration. J. Immunol..

[34] Cellier E., Midaoui A.E., Robitaille G.A., Richard D.E., Lariviere R., Lebel M. (2010). Effects of TGF-beta1 on endothelial factors. Arch. Physiol. Biochem..

[35] Zhang R., Oyajobi B.O., Harris S.E., Chen D., Tsao C., Deng H.W., Zhao M. (2013). Wnt/β-catenin signaling activates bone morphogenetic protein 2 expression in osteoblasts. Bone.

[36] Liu H., Zhang R., Chen D., Oyajobi B.O., Zhao M. (2012). Functional redundancy of type II BMP receptor and type IIB activin receptor in BMP2-induced osteoblast differentiation. J. Cell. Physiol..

[37] Mandal C.C., Ganapathy S., Gorin Y., Mahadev K., Block K., Abboud H.E., Harris S.E., Ghosh-Choudhury G., Ghosh-Choudhury N. (2011). Reactive oxygen species derived from Nox4 mediate BMP2 gene transcription and osteoblast differentiation Chandi. Biochem. J..

[38] Lazzeri L., Cascone M.G., Danti S., Serino L.P., Moscato S., Bernardini N. (2006). Gelatine/PLLA sponge-like scaffolds: Morphological and biological characterization. J. Mater. Sci. Mater. Med..

[39] Mattii L., Battolla B., D’Alessandro D., Trombi L., Pacini S., Cascone M.G., Lazzeri L., Bernardini N., Dolfi A., Galimberti S. (2008). Gelatin/PLLA sponge-like scaffolds allow proliferation and osteogenic differentiation of human mesenchymal stromal cells. Macromol. Biosci..

[40] Trombi L., D’Alessandro D., Pacini S., Fiorentino B., Scarpellini M., Fazzi R., Galimberti S., Guazzini S., Petrini M. (2008). Good manufacturing practice-grade fibrin gel is useful as a scaffold for human mesenchymal stromal cells and supports in vitro osteogenic differentiation. Transfusion.

[41] Trombi L., Mattii L., Pacini S., D’alessandro D., Battolla B., Orciuolo E., Buda G., Fazzi R., Galimberti S., Petrini M. (2007). Human Autologous Plasma-Derived Clot as a Biological Scaffold for Mesenchymal Stem Cells in Treatment of Orthopedic Healing. J. Orthop. Res..

[42] Danti S., Stefanini C., D’Alessandro D., Moscato S., Pietrabissa A., Petrini M., Berrettini S. (2009). Novel biological/biohybrid prostheses for the ossicular chain: Fabrication feasibility and preliminary functional characterization. Biomed. Microdevices.

[43] Mercado-Pagàn A.E., Stahl A.M., Shanjani Y., Yang Y. (2016). Vascularization in bone tissue engineering constructs. Ann. Biomed. Eng..

[44] Checchi M., Smargiassi A., Ferretti M., Sena P., Benincasa M., Cavani F., Ranieri A., Mitola S., Palumbo C. (2016). Preliminary observations on scleral ossicles in performing functionalized 3D vascularized scaffolds for “critical-size” bone defect healing. Ital. J. Anat. Embryol..

[45] Checchi M., Grisendi G., Bertacchini J., Magarò M.S., Ferretti M., Benincasa M., Sena P., Cavani F., Palumbo C. (2017). Scleral ossicles as natural biomaterials on which vascular-like network is promoted from Mouse Aortic Endothelial cells ( MAECs ): Preliminary results. Ital. J. Anat. Embryol..

[46] Kuss M.A., Wu S., Wang Y., Untrauer J.B., Li W., Lim J.Y., Duan B. (2017). Prevascularization of 3D printed bone scaffolds by bioactive hydrogels and cell co-culture. J. Biomed. Mater. Res. Part B Appl. Biomater..

[47] Zhang S., Zhou M., Ye Z., Zhou Y., Tan W.S. (2017). Fabrication of viable and functional pre-vascularized modular bone tissues by coculturing MSCs and HUVECs on microcarriers in spinner flasks. Biotechnol. J..

